# 3D-motion mapping of the malleus–incus complex using a robot-mounted optical coherence tomography vibrometry system

**DOI:** 10.1117/1.JBO.31.12.123303

**Published:** 2026-02-20

**Authors:** Nathan Goedseels, Pieter Livens, Yang Li, Guy Fierens, Nicolas Verhaert, Tristan Putzeys

**Affiliations:** a KU Leuven, Medicine, Neurosciences, Leuven, Belgium; b University Hospitals Leuven, Otorhinolaryngology, Head and Neck Surgery, Leuven, Belgium; c University of Antwerp, Biophysics and Biomedical Physics, Antwerp, Belgium; d Cochlear Technology Centre Belgium, Mechelen, Belgium; e KU Leuven, Department of Physics and Astronomy, Laboratory for Soft Matter and Biophysics, Leuven, Belgium

**Keywords:** optical coherence tomography, vibrometry, malleus–incus complex, middle ear mechanics

## Abstract

**Significance:**

Optical coherence tomography vibrometry (OCTv) allows measuring the surface and subsurface nanometer vibrations of the mammalian ossicular chain. However, existing multidimensional OCTv setups remain expensive, complex, or limited in accuracy.

**Aim:**

We developed a 3D OCTv setup with a rotational component and provided a theoretical framework that clarifies the main determinants of measurement accuracy in 3D vibrometry.

**Approach:**

A commercially available OCTv system is mounted on a robotic arm to allow multidimensional measurements. The relative positions of measured structures and the optical axes orientation are defined with a custom volume registration algorithm.

**Results:**

We present a mathematical framework for decomposing the measured motion components into a Cartesian space and identify key factors that influence the decomposition accuracy. The angular accuracy of optical axis estimation was 0.4 deg. Experimental validation was performed on an oscillating phantom and on the malleus–incus complex (MIC) of a fresh human temporal bone specimen, replicating previous evidence on the MIC’s frequency-dependent vibratory behavior.

**Conclusions:**

The robot-mounted 3D OCTv setup provides a cost-effective, robust, and integral solution for mapping middle ear 3D vibrations, accurately orienting optical axes across measurements. Future work should integrally map the ossicular chain to test the commonly assumed rigid-body behavior of ossicular motion.

## 1 Introduction

The tympanic membrane and the ossicular chain are essential for sound perception, allowing the impedance matching of sound waves travelling through air in the external ear canal and fluid in the inner ear. This transfer of acoustic energy partly relies on the 3D vibrations of the malleus–incus complex (MIC) that consists of the malleus, the incus, and the incudomalleolar joint, especially important for perceiving higher frequencies.^1^^,^^2^

For decades, laser doppler vibrometry (LDV) has been used to measure these nano-vibrations.^3^^,^^4^ However, LDV only reports vibrations of the dominant reflector along the beam path and therefore requires a precisely focused beam, most often on a strongly reflective structure that is applied to the object of interest. More recently, optical coherence tomography (OCT) and OCT vibrometry (OCTv) allow noninvasive imaging at depths below the tissue surface with the vibratory response available at the pixel level.^5^^–^^7^ In addition, OCT only requires coarse positioning of the sample for it to be situated within the OCT’s optical depth of focus. For a spectral-domain OCT system, imaging is based on the raw linear image sensor data of back-scattered light of a one-dimensional (1D) axial scan that is Fourier transformed to a depth-profile, or A-scan, which contains both a magnitude and a phase. The magnitude of the A-scan corresponds to the reflectivity of a structure at a certain depth into the tissue. Hence, structures along the beam path can be discriminated, forming the OCT image.^5^^,^^8^ Multiple A-scans along a line perpendicular to the optical axis can be combined into a cross-sectional (2D) B-scan. In turn, multiple parallel B-scans can be structured into a volumetric (3D) C-scan.

In OCTv, the phase of the A-scan is used to spatially resolve the displacement of the measured tissue to an acoustic stimulus. More specifically, for a series of A-scans with a stationary beam location, the phase of a pixel over time is proportional to the sub-pixel displacement along the optical axis of the structure at that pixel.^9^ In the OCT literature, these A-scans over time are referred to as an M-scan (motion-scan).^9^^,^^10^ These M-scans can be systematically repeated at different beam locations as described above to obtain a cross-sectional M-scan and a volume M-scan.^8^^,^^10^^,^^11^

However, the complex 3D vibratory patterns of the MIC can only be elucidated with measurements from multiple directions. Earlier work achieved multidimensional OCTv in several ways. A higher degree optical system allows multidimensional measurements but comes at the cost of increased optical complexity. This has been achieved by integrating multiple separate OCT systems or by adapting a single OCT system for multidimensional use with a beam divider in the sample arm.^12^^–^^14^ Moreover, Kim et al. built a custom OCT system that combined three sample arms into a single interferometer, allowing simultaneous multidimensional measurements and thereby significantly decreasing data acquisition time.^10^

In these higher-degree optical setups, the directions of the optical axes are restrained to the optical setup. This allows an accurate calibration of the optical axes, paramount for the decomposition of the measured components into a Cartesian space, also termed the “orthogonal decomposition.” However, such fixed optical axes are undesirable in the context of hearing mechanics, often dealing with limited optical access to the tissue.^10^ The introduction of a rotational component to a single-beam set-up—with the rotational element being either the scanning system or the specimen—allows measurements from any direction within the range of rotation. Nevertheless, it requires serial multidimensional measurements, increasing experimental complexity, and complicates the precise definition of the optical axes. Lee et al. and Frost et al. achieved two-dimensional OCTv measurements in a rodent cochlea by carefully rotating the rodent head between measurements.^9^^,^^15^^,^^16^ The authors elegantly defined the optical axes based on anatomical and physiological properties of the cochlea. Although valuable methods, the optical axes are extracted with manual selection of points in proximity to an anatomical structure. Such a vicinity-based approach carries an inherent directional uncertainty in the estimated optical axis. Frost et al. showed that a realistic within-user variability can result in a variability in optical axis estimation up to 15 deg.^15^ In addition, specimen rotation is prone to motion artefacts when measurements are performed before a new equilibrium is reached or leads to shifts in vibration patterns due to altered gravitational force on the object. Finally, motion-induced shifts in the position of the sound source within the ear canal can introduce phase errors, further complicating data interpretation.

Although calibration of the optical axes is one factor, the accuracy of the orthogonal decomposition is also influenced by noise amplification. The orthogonal decomposition corresponds to a linear combination of at least three measured motion components along different optical axes. This linear combination also applies to any noise contained in the measurements that can consequently be magnified.^9^^,^^17^ Therefore, the selection of the optical axes must account for the limited optical access to the ossicular chain while keeping the level of noise amplification within reasonable limits.

Here, we introduce a 3D OCTv set-up with a commercially available spectral domain OCT scanning system mounted on a 6 degrees of freedom robot arm. The use of a single-beam OCTv limits optical complexity and is cost-effective, whereas the rotational component introduces a critical versatility to the setup, mitigating the variable and often limited optical access to middle ear structures. The calibration of the optical axis is achieved with a custom volume registration algorithm, which has proven to be a valuable method for OCT data.^18^ In addition, as the impact of optical axis estimation and noise amplification on decomposition accuracy is often rather ill-defined, we provide and experimentally validate a theoretical framework to understand and manage both factors. Finally, we demonstrate that our method is suitable for research in middle ear mechanics with *ex vivo* experimental data on the MIC of a fresh human temporal bone specimen, replicating previous evidence on the MIC’s frequency-dependent vibratory behavior.

## 2 Materials and Methods

### 2.1 Imaging System and Robotic Arm

**Fig. 1:**
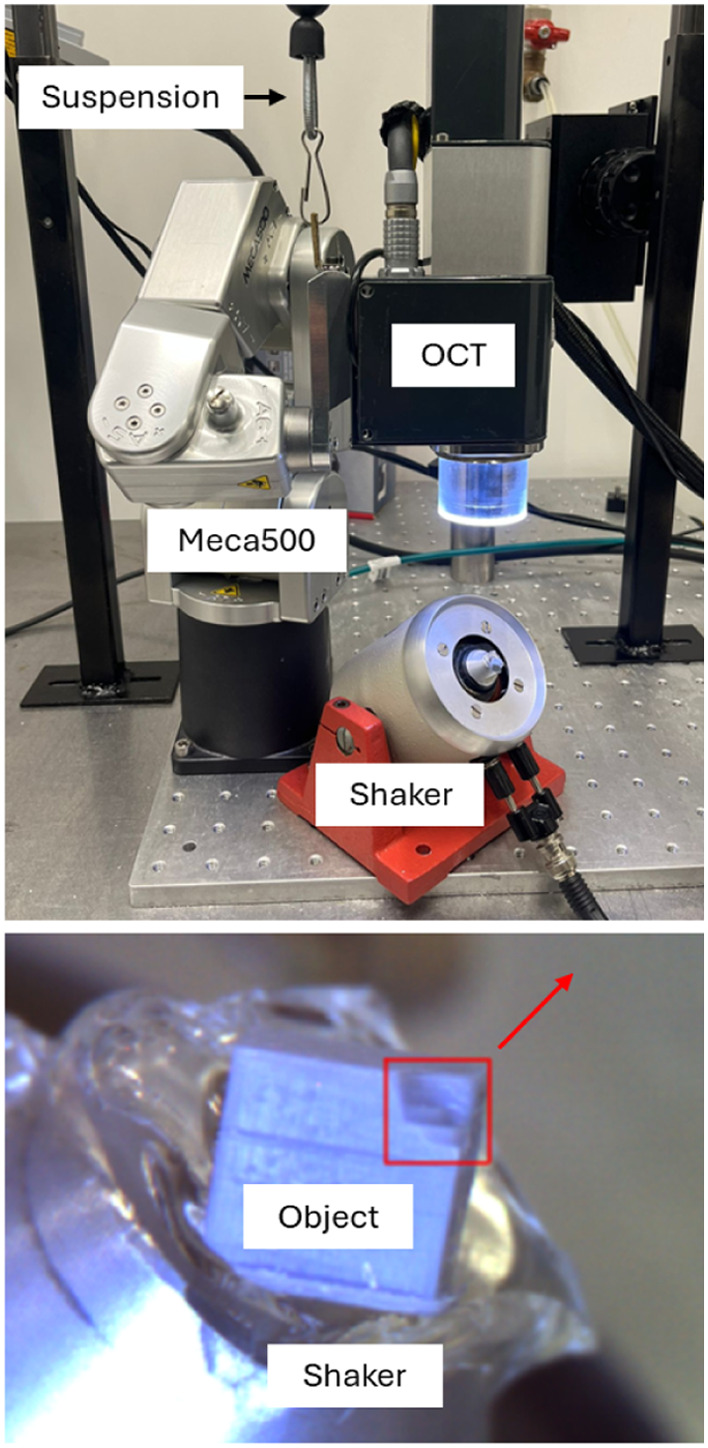
Imaging system, robotic arm with spring balancer (suspension), and shaker with object. The red square denotes the stair-like features; the red arrow denotes the direction of vibration.

A commercially available spectral-domain OCT system with a central wavelength of 1310 nm (Thorlabs, TEL321C1, Newton, New Jersey, United States) and lens (Thorlabs, LSM04) were used for all measurements. The OCT system was mounted with a custom L-bracket on a six degrees of freedom robotic arm, the Meca500 (Mecademic, Montreal, Canada). The robotic arm was connected to a spring balancer attached to two elevated mobile rails so that the spring followed the robotic arm’s position in an elevated horizontal plane, ensuring the spring force to be along the vertical at all times. Before the start of a measurement, the net force on the robotic arm was minimized by manually adjusting the spring winding so that the gravitational force of the OCT system was counterbalanced by the spring force (Fig. 1). The robotic arm allows positioning the OCT scanning system at a desired position with high precision such that measurements can be performed from any direction, referred to as a direction measurement (DM).

### 2.2 Phantom Object

All measurements were performed on a solid 3D-printed object (polymethylmethacrylate, PMMA, refractive index = 1.4906) with asymmetrical stair-like features mounted on a tilted shaker (Type 4810, Brüel & Kjær, Denmark). The object features were specifically designed to prevent shadow casting or full penetration of the light under any condition.

We assume that the rigid object vibrates uniformly such that the amplitude and phase at any point on the surface or below are equal. However, a scan contains complex Gaussian noise. Therefore, the measured amplitudes are normally distributed around the true vibration amplitude $A_{t}$, which corresponds to the mean of the vibrometric measurement. This assumption was validated with an LDV measurement as a reference (Fig. S1 in the Supplementary Material).

### 2.3 Sound-Synchronous Stimulus Protocol

As multiple OCTv dimensionalities are used alternately throughout this paper, an altered terminology is used. An M-scan, cross-sectional M-scan, and volume M-scan are termed $M_{A}$-scan, $M_{B}$-scan, and $M_{C}$-scan, respectively—in analogy to the dimensions of the OCT magnitude images.

The phase difference Δϕ of a pixel in two successive A-scans is proportional to the subpixel displacement along the optical axis, or axial displacement, of the corresponding reflective structure:^9^^,^^19^

$$\Delta \phi =\frac{4\pi }{\lambda }\times \Delta z,$$ (1)

where $\Delta \phi =\phi (t_{2})-\phi (t_{1})$, $\Delta z=z(t_{2})-z(t_{1})$, and λ is the center wavelength of the OCT light source. The instantaneous axial velocity v can be approximated by v=Δz/Δt, so therefore

$$v=\frac{\lambda \Delta \phi }{4\pi \Delta t},$$ (2)

where $\Delta t=t_{2}-t_{1}$ is the time interval between two A-scans.^19^

**Fig. 2:**
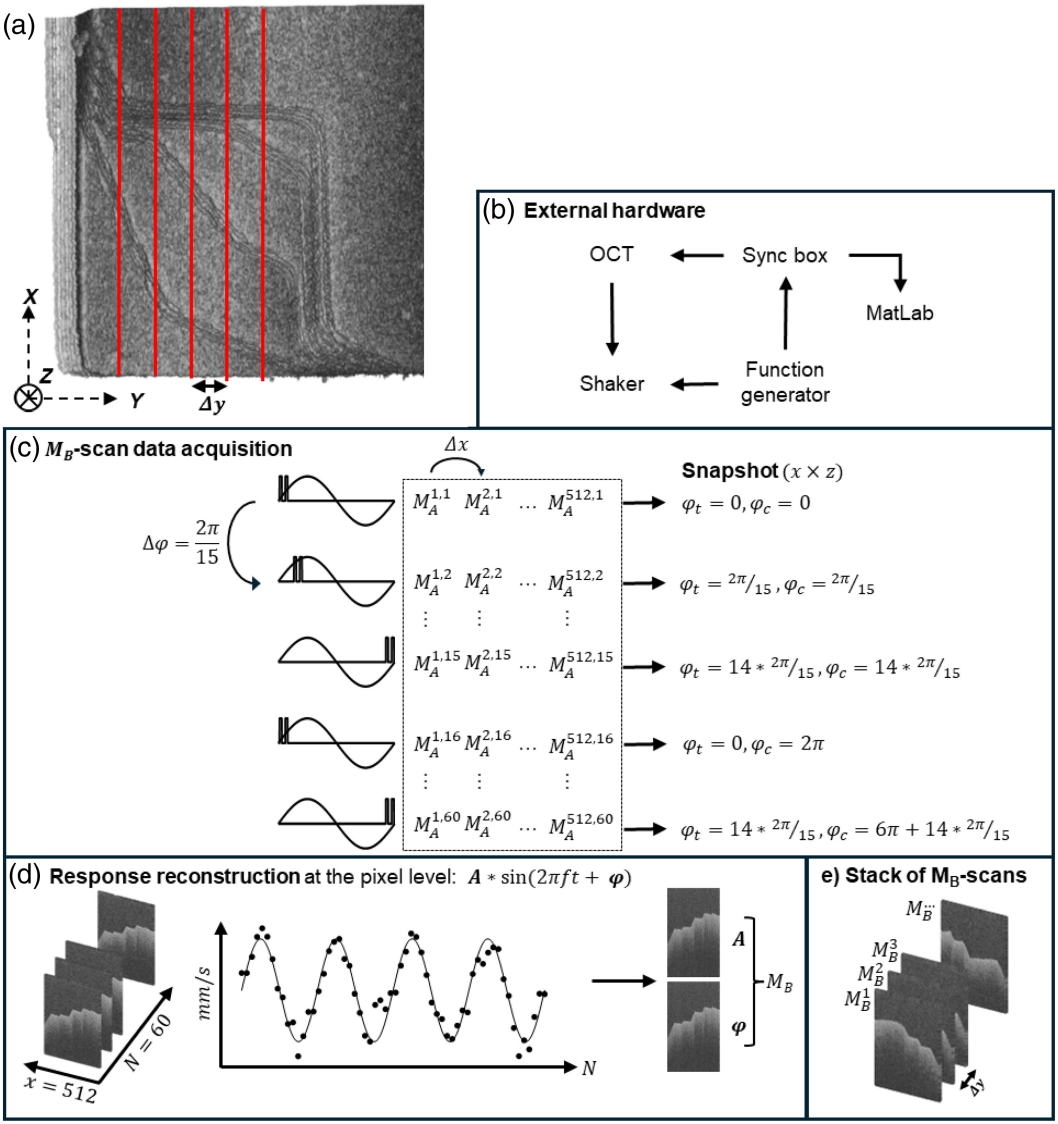
(a) Superior view of a C-scan with superimposed $M_{B}$-scan directions (red lines). Axes of the OCS are shown. The z-axis corresponds to the optical axis. (b) External hardware to synchronize A-scan triggering with the stimulus to calculate velocity. (c) The $M_{B}$-scan data acquisition process based on serial $M_{A}^{x,N}$ scans. The dotted rectangle comprehends the entire $M_{B‐\text{scan}(x\times z\times N)}$. (d) Pixel-level response reconstruction (sine wave) from the obtained instantaneous velocities (points). The sine wave can be expressed as its amplitude A and its phase φ for all pixels. (e) After completion of an $M_{B}$-scan, a Δy is introduced, and the process repeats, resulting in a stack of $M_{B}$-scans. For visualization purposes, the B-scans are shown. OCS, optical coordinate system; $\varphi_{t}$, trigger phase; $\varphi_{c}$, cumulative phase.

Figure 2 shows the data collection protocol. ThorImage software (v5.7.1) from Thorlabs is used with external trigger mode activated, i.e., for every trigger received, an A-scan is performed. A function generator (SDG1032X, Siglent, Shenzhen, China) generates a sine stimulus with a frequency of 1000 Hz and a trigger at π=0 in separate channels. The stimulus channel is used to drive the shaker. The trigger channel is connected to a custom trigger synchronization box controlled via MATLAB (R2024b Mathworks, Massachusetts, United States) that is in turn connected to the OCT system.

The data acquisition and beam scanning are arranged such that at every transverse location [x,y], 60 phase differences Δϕ are measured, synchronized to the stimulus at 60 phases φ over four cycles. Note that the notation ϕ corresponds to the OCT phase, whereas φ relates to the phase of the shaker stimulus or the consequent object oscillation. For each received trigger, the synchronization box generates two TTL triggers separated by a Δt=9  μs that are routed to the OCT system. These triggers initiate two serial A-scans, each size (1×z), at Δt=9  μs at the beam location. The phase difference Δϕ of these serial A-scans corresponds to an $M_{A}$-scan (1×z), which must be distinguished from a single A-scan despite equal dimensionality. After completing the $M_{A}$-scan, the OCT beam steps to the next lateral location, Δx. This process is repeated 512 times to construct a “snapshot,” size (x×z), of the sample at a single phase of the oscillating object.^8^^,^^11^ Next, the phase of the first output trigger $\varphi_{t}$ from the synchronization box is advanced by Δφ=2π/15, and the process is repeated until a total of N=60 snapshots are acquired, (x×z×N). Importantly, $\varphi_{t}$ is wrapped to the interval [0,2π], such that for snapshot N=16, $\varphi_{t}=0$. Every snapshot corresponds to a cumulative phase $\varphi_{c}=(N-1)\times (2\pi /15)$. By applying a correlation filter at the stimulus frequency, the sinusoidal response of the oscillating object to the input stimulus can be reconstructed at the pixel level (x,z) to obtain an $M_{B}$-scan. That is, for every pixel (x,z), the oscillatory response is reconstructed from 60 instantaneous axial velocities v, calculated from the measured phase differences Δϕ [Eq. (2)] [Fig. 2(d)]. Importantly, these 60 instantaneous axial velocities stem from the pair-wise phase differences of two serial A-scans, as described above, rather than the phase difference of an entire array of 60 A-scans, which would result in only 59 velocity calculations. From this $M_{B}$-scan, a cross-sectional amplitude A=|v| and phase φ=∠v map can be obtained, with vibratory information available at the pixel level. In addition, a B-scan can be obtained from the $M_{B}$-scan by taking the magnitude of the depth-resolved interference signal of a single snapshot. Finally, the scanned cross-section is altered with an automated shift, Δy, to construct a new $M_{B}$-scan. This is repeated to obtain a stack of $M_{B}$-scans [Fig. 2(e)]. The presented data acquisition protocol differs from other papers, where most often the beam is kept stationary throughout a single stimulus period, whereas A-scans are measured at a fixed sampling frequency before advancing the beam to the next lateral position.^8^^,^^11^

### 2.4 Construction of M_C_-scans

The ThorImage software allows only C-scans and, when combined with the external triggering hardware, $M_{B}$-scans. Therefore, an $M_{C}$-scan is constructed by combining multiple $M_{B}$-scans.

**Fig. 3:**
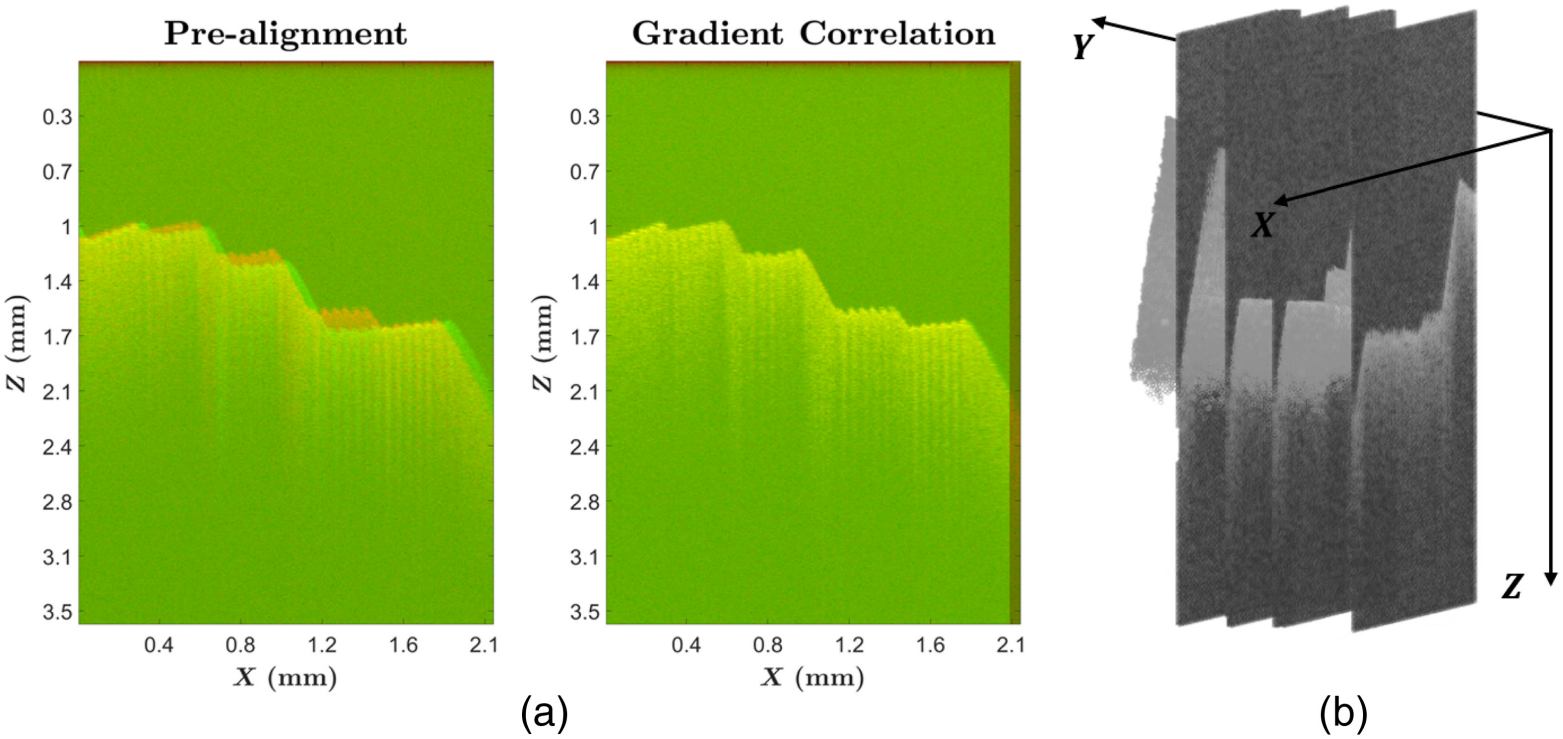
(a) $M_{B}$-scan (red) superimposed with a C-scan slice (green). Left: pre-alignment based on internal calibration parameters showing a shift. Right: alignment after gradient correlation resulting in a quasi-perfect overlap. (b) Stack of $M_{B}$-scans positioned within the background-subtracted C-scan.

For every DM, a C-scan and a stack of $M_{B}$-scans were obtained. $M_{B}$-scans are aligned with the corresponding cross-section of the C-scan in two steps. First, the internal calibration parameters of the scanning system are used for pre-alignment. Second, a slice-to-slice registration using gradient correlation allows a robust alignment algorithm that provides 3D translation components (Fig. 3).

After alignment, a background subtraction is performed for both the B-scans and the C-scan using a reflectivity threshold of 50 dB, empirically determined for this specific setup. The background-subtracted B-scan was used as a mask for the $M_{B}$-scan. Finally, the vibratory information of the aligned and background-subtracted $M_{B}$-scans is interpolated to the mask of the background-subtracted C-scan to construct the $M_{C}$-scan. The interpolation is achieved with a scattered interpolation method with nearest neighbor interpolation and no extrapolation. ChatGPT (OpenAI) was used to facilitate code writing with prompts such as: “code a gradient correlation optimization problem between slices”. All AI-generated content was carefully reviewed and edited by the authors.

### 2.5 Mathematics for the Orthogonal Decomposition

In the world coordinate system (WCS), the velocity vector v has Cartesian components $v_{x}$, $v_{y}$, and $v_{z}$ along axes $x_{w}$, $y_{w}$, and $z_{w}$. The optical axis d is a unit vector with three components $d_{x}$, $d_{y}$, and $d_{z}$. For an n’th DM with optical axis $d^{n}$, the measured projection $v_{n}$ of v onto the optical axis in the WCS can be written as:

$$v^{n}=v.d^{n}=v_{x}d_{x}^{n}+v_{y}d_{y}^{n}+v_{z}d_{z}^{n.}$$ (3)

Importantly, $v_{n}\in C$ corresponds to periodic motion such that it has a magnitude and a phase. If we consider three DMs with different optical axes:

$$v^{1}=v.d^{1},\;v^{2}=v.d^{2},\;v^{3}=v.d^{3}.$$ (4)

We can rewrite Eq. (4) as a matrix equation, assuming all displacements are sampled coherently, and each DM observes the same physical displacement field:

$$[\begin{array}{c} v_{1}\\ v_{2}\\ v_{3} \end{array}]=[\begin{array}{ccc} d_{x}^{1} & d_{y}^{1} & d_{z}^{1}\\ d_{x}^{2} & d_{y}^{2} & d_{z}^{2}\\ d_{x}^{3} & d_{y}^{3} & d_{z}^{3} \end{array}]\times [\begin{array}{c} v_{x}\\ v_{y}\\ v_{z} \end{array}].$$ (5)

The optical coordinate system (OCS) is defined by the OCT scanning system, where $z_{l}$ corresponds to the optical axis, $x_{l}$ is the direction of lateral scanning and $y_{l}$ is perpendicular to $x_{l}$ (Fig. 1). Let us define the WCS as the OCS of the first of the three measurements, or the reference measurement, such that its optical axis $z_{l}$ corresponds to $z_{w}=[0\;0\;1{]}^{T}$. If we solve for the Cartesian components:

$$[\begin{array}{c} v_{x}\\ v_{y}\\ v_{z} \end{array}]=[\begin{array}{ccc} 0 & 0 & 1\\ d_{x}^{2} & d_{y}^{2} & d_{z}^{2}\\ d_{x}^{3} & d_{y}^{3} & d_{z}^{3} \end{array}{]}^{-1}\times [\begin{array}{c} v_{1}\\ v_{2}\\ v_{3} \end{array}],$$ (6)

where $O=[z_{w},d^{2},d^{3}{]}^{T}$. Equation (6) is the orthogonal decomposition. O must be invertible, which requires the three optical axes to be linearly independent. This condition can be ensured via the selection of the optical axes of the different DMs. We then express the second and third optical axes in spherical coordinates in the WCS:

$$[\begin{array}{c} v_{x}\\ v_{y}\\ v_{z} \end{array}]=[\begin{array}{ccc} 0 & 0 & 1\\ \sin\;\theta_{1}\;\cos\;\varphi_{1} & \sin\;\theta_{1}\;\sin\;\varphi_{1} & \cos\;\theta_{1}\\ \sin\;\theta_{2}\;\cos\;\varphi_{2} & \sin\;\theta_{2}\;\sin\;\varphi_{2} & \cos\;\theta_{2} \end{array}{]}^{-1}\times \;[\begin{array}{c} v_{1}\\ v_{2}\\ v_{3} \end{array}].$$ (7)

Let A be a 3×3 matrix, then $A^{-1}=\mathrm{adj}(A)/\det(A)$. The determinant of O corresponds to:

$$\det(O)=\sin\;\theta_{1}*\sin\;\theta_{2}*\sin(\varphi_{2}-\varphi_{1}).$$ (8)

This highlights the singularities where the inversion of O becomes ill-conditioned: for $\theta_{1}$ and $\theta_{2}$ approaching zero, e.g., when the two non-reference axes are too close to the reference axis, or for $\Delta \varphi =\varphi_{2}-\varphi_{1}$ approaching zero, when the axes are too close together in azimuth.

### 2.7 Decomposition Accuracy Degrading Factors

#### 2.7.1 Noise amplification

The complex OCTv signal contains detector noise, which is additive circular zero-mean Gaussian noise and should be distinguished from the multiplicative speckle noise present in OCT magnitude images.^7^ This additive noise can be amplified through the orthogonal decomposition.^9^^,^^10^^,^^17^ Writing the OCTv signal of an i’th measurement as $v_{i}+n_{i}$, with $n_{i}$ being the noise term, the noise terms found after Eq. (6) correspond to:

$$\left\{ \begin{array}{c} n_{x}=\;O_{11}^{-1}\times n_{1}+O_{12}^{-1}\times n_{2}+O_{13}^{-1}\times n_{3}\\ n_{y}=O_{21}^{-1}\times n_{1}+O_{22}^{-1}\times n_{2}+O_{23}^{-1}\times n_{3}\\ n_{z}=O_{31}^{-1}\times n_{1}+O_{32}^{-1}\times n_{2}+O_{33}^{-1}\times n_{3} \end{array},\right.$$ (9)

where $n=(n_{1},n_{2},n_{3})$ are the complex noise parameters contained in the DMs and $n^{\left(d\right)}=(n_{x},n_{y},n_{z})$ contains the decomposed complex noise parameters. Due to the co-alignment of the reference optical axis with $z_{w}$, $n_{z}$ reduces to $n^{1}$ [Eq. (9)].

As the noise is primarily additive, the noise distribution does not depend on the vibration amplitude. Consequently, given the same scanning system and data processing was used for all DMs, the noise distribution can be assumed to be approximately equal between DMs, regardless of the measured vibration amplitude. Therefore, Eq. (9) can be approximated as a linear combination of mutually independent, normal random variables with equal distribution $\left(\sigma_{n^{1}}\approx \sigma_{n^{2}}\approx \sigma_{n^{3}}\approx \sigma_{n}\right)$ such that the standard deviation of the decomposed noise corresponds to:

$$\sigma_{n_{x}}\approx \sqrt{O_{11}^{-12}+O_{12}^{-12}+O_{13}^{-12}}\times \sigma_{n}=\left\|O_{1,.}^{-1}\right\|\times \sigma_{n},$$ (10)

$$\sigma_{n_{y}}\approx \sqrt{O_{21}^{-12}+O_{22}^{-12}+O_{23}^{-12}}\times \sigma_{n}=\|O_{2,.}^{-1}\|\times \sigma_{n},$$ (11)

where $\sigma_{n}^{\left(d\right)}=(\sigma_{n_{x}},\sigma_{n_{y}})\;$ contains the distributions of the decomposed noise parameters. We define a noise amplification factor (NAF) such that $NAF_{x}=\|O_{1,.}^{-1}\|$ and $NAF_{y}=\|O_{2,.}^{-1}\|$. In practice, high values of NAF indicate greater sensitivity of the decomposition to measurement noise. This highlights the importance of optimizing the relative orientation between DMs to minimize NAF and maintain decomposition accuracy.

Importantly, an increase in sample reflectivity, i.e., OCT magnitude, improves the system sensitivity.^7^ Hence, $\sigma_{n}$ depends on the threshold used for the background subtraction described above and on the reflectivity decay along the depth dimension, i.e., the sample composition.

#### 2.7.2 Noise floor estimation

The magnitude A=|v| of a complex Gaussian signal follows a Rician distribution. Only in the high signal-to-noise ratio $A_{t}/\sigma_{n}\gg 1$ regime, where $A_{t}$ is the true vibration amplitude and $\sigma_{n}$ is the standard deviation of the complex noise contained in the signal, the Rician distribution of A approximates a Gaussian centered at $A_{t}$ with variance approximately equal to $\sigma_{n}^{2}$. Provided that

$$A_{t}/\sigma_{n}>3,$$ (12)

the nonlinear amplitude mapping of v to A preserves the Gaussian distribution.^7^^,^^20^^,^^21^ If the condition in Eq. (13) is not met, the distribution in A is skewed by Rician bias, leading to an overestimation of the amplitude. Hence, a measure for the system’s noise floor, the lowest reliably detectable vibration amplitude, can be derived from Eq. (12):

$$A_{\min}=3*\sigma_{n}.$$ (13)

For a given orthogonal decomposition, the noise statistics in the DMs are preserved after orthogonal decomposition if for each Cartesian component:

$$\frac{A_{t}^{\left(d\right)}}{\sigma_{n}^{\left(d\right)}}>3,$$ (14)

where $A_{t}^{\left(d\right)}=(A_{t_{x}},A_{t_{y}})$ are the true decomposed vibration amplitudes along $x_{w}$ and $y_{w}$, respectively. Hence, according to Eqs. (10), (11) and (13)–(15), the noise floor after the orthogonal decomposition is defined by

$$A_{\min}^{\left(d\right)}=3\times {\mathrm{NAF}}_{\max}\times \sigma_{n},$$ (15)

where $A_{\min}^{\left(d\right)}$ is the decomposition-specific noise floor and ${\mathrm{NAF}}_{\max}$ corresponds to the largest of both NAFs.

On the other hand, in the high SNR (>3) regime, the distribution of the nonlinear phase mapping φ=∠v is inversely related to the signal amplitude, with the standard deviation approximated by Eq. (16), assuming small phase variance and unwrapped phase:^21^

$$\sigma_{\varphi }=\frac{\sigma_{n}}{A_{t}}.$$ (16)

At higher vibration amplitudes, the dominant noise source shifts from additive detector noise to motion-dependent sources associated with timing jitter and the finite temporal sampling. However, in this high-amplitude regime, explicit noise-floor estimation becomes less relevant as the signal can be assumed to lie well above the (increased) noise floor so that Rician bias is not introduced and the ossicular chain vibrations can be reliably measured with OCTv, even under superphysical stimulation intensities.

#### 2.7.3 Estimation of the optical axes

The accurate estimation of the optical axis is crucial for the accuracy of the orthogonal decomposition. The optical axes are defined by a custom volume registration Python pipeline to improve the robotic arm’s empirically measured angular error of ∼3  deg. Here, the surface points of the scanned object in the C-scans are extracted, and the obtained surfaces are aligned using random sample consensus and the iterative closest point as measures.^22^^,^^23^ This alignment is performed twice for an orthogonal decomposition, where the C-scans of the two non-reference DMs are registered to the C-scan of the reference DM. From both registrations, a 4×4 transformation matrix R is obtained, one for each registration. The estimated optical axes $d^{2}$ and $d^{3}$ correspond to the respective rotated $z_{l}$, the first three rows of the third column of the respective R.

**Fig. 4:**
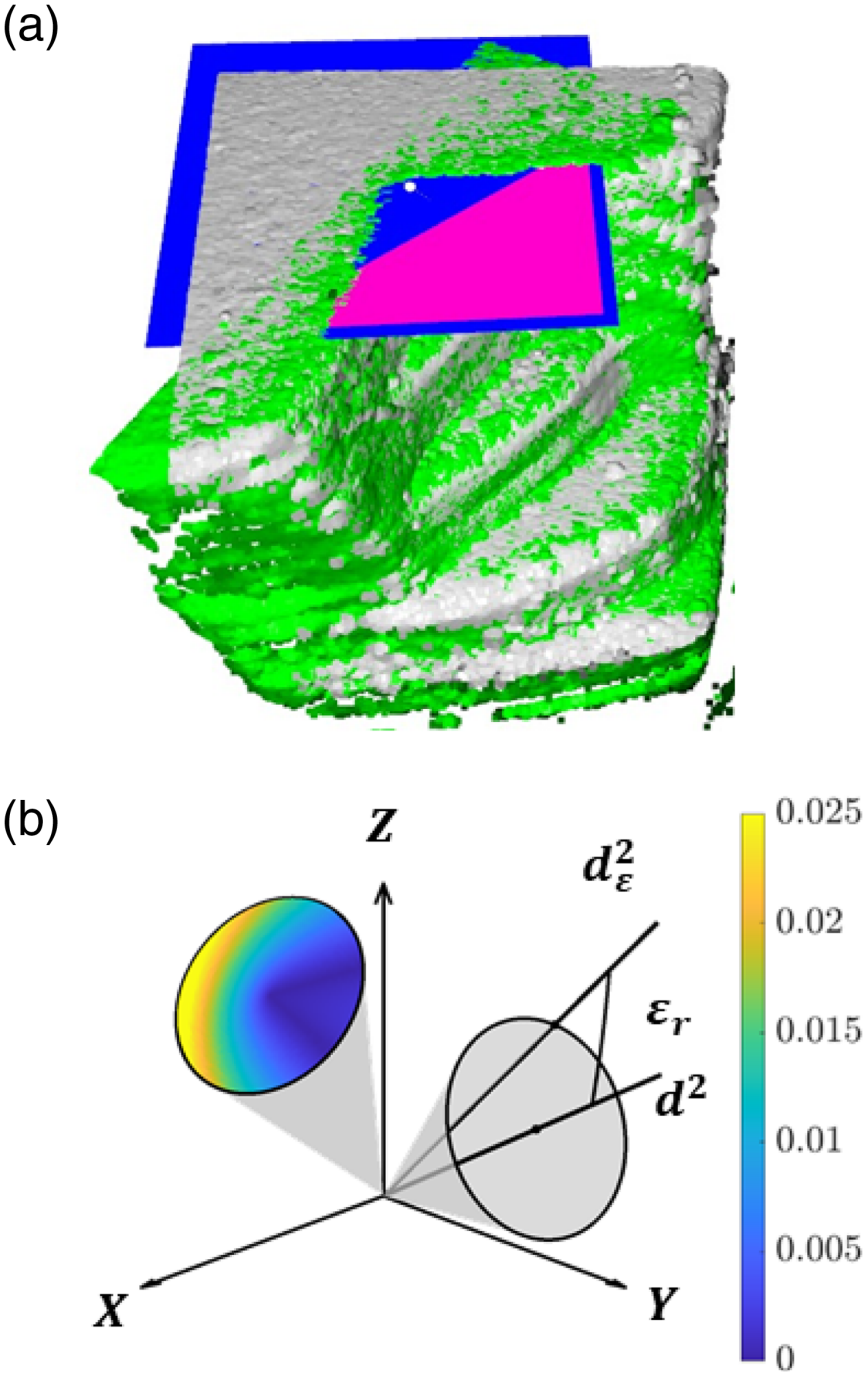
(a) Surface point clouds of the reference C-scan (grey) and the moving C-scan (green) used for the volume registration with the intersecting horizontal surface planes (blue and pink). (b) Two conical uncertainty regions for arbitrarily chosen beam axes $d^{2}(\theta =25\;\deg,\varphi =30\;\deg)$ and $d^{3}(\theta =20\;\deg,\varphi =40\;\deg)$ with $\varepsilon_{r}=0.4\;\deg$. In color, the decomposition error $\varepsilon_{d_{x}}$ from components $v_{1}=v_{2}=v_{3}=1+0i$, for a given $d_{\varepsilon }^{2}$ and all $d_{\varepsilon }^{3}$.

The quality of the decomposition thus depends on the quality of the registration algorithm. A feature-based method is used to estimate its accuracy. Here, a plane is fitted through the upper horizontal surfaces of both registration volumes of the phantom object. For a perfect registration, both planes coincide. Any deviation due to suboptimal registration results in an intersection. The angle between the intersecting planes is defined as the registration error $\varepsilon_{r}$ [Fig. 4(a)]. According to this evaluation metric, the algorithm achieves registrations with an estimated $\varepsilon_{r}$ of 0.4  deg±0.1  deg (Fig. S2 in the Supplementary Material).

This registration error $\varepsilon_{r}$ is applicable for both non-reference DMs in the orthogonal decomposition. The decomposition matrix $O_{\varepsilon }^{-1}$, constructed from the estimated (and potentially erroneous) optical axes, is used in the inverse operation to compute the Cartesian motion components. Errors in axis estimation propagate through this inversion, causing deviations in the decomposed Cartesian components. The differences between the resulting erroneous Cartesian components $v_{x_{\varepsilon }}$ and $v_{y_{\varepsilon }}$, and $v_{x}$ and $v_{y}$, correspond to the decomposition error $\varepsilon_{d_{x}}$ and $\varepsilon_{d_{y}}$, respectively, with each an amplitude and phase component.

Importantly, the amplitude component of $\varepsilon_{d_{x,y}}$ can differ significantly for a given $\varepsilon_{r}$. To illustrate this, $\varepsilon_{r}$ can be modelled as a conical uncertainty region centered on the true beam axis $d^{n}$ with a semi-vertical angle equal to $\varepsilon_{r}$. For a given $d_{\varepsilon }^{2}$, an infinite amount of $d_{\varepsilon }^{3}$ exists to perform the orthogonal decomposition. The final amplitude decomposition error depends heavily on the orientation of both $d_{\varepsilon }^{2}$ and $d_{\varepsilon }^{3}$ relative to their respective true optical axis [Fig. 4(b)]. It is therefore impossible to accurately predict the amplitude decomposition error despite a good estimate of the registration error. As a rule-of-thumb, the inversion of an ill-conditioned O is more sensitive to alignment errors and results in higher amplitude decomposition errors. This highlights the importance of bringing $\theta_{1}$, $\theta_{2}$, and Δφ as close to π/2 as experimentally possible [Eq. (8)]. Consequently, NAF is a measure for both the amplified noise floor due to noise amplification [Eq. (15)] as for the amplitude decomposition error.

### 2.8 Orthogonal Decomposition on the Voxel Level

In addition to estimating the optical axes, the volume registration algorithm allows the definition of voxels between DMs at approximately the same point in space. The transformation matrix obtained from the volume registration is applied as a coordinate transform, mapping each DM onto the WCS grid via inverse scattered interpolation (nearest neighbor, no extrapolation). This aligns the geometry without altering velocity components, enabling the voxel-level orthogonal decomposition. Importantly, orthogonal decomposition can only occur at voxels with information from at least three DMs. With less than three velocity components, the system cannot be locally solved.

### 2.9 Phantom Direction Measurements

**Fig. 5:**
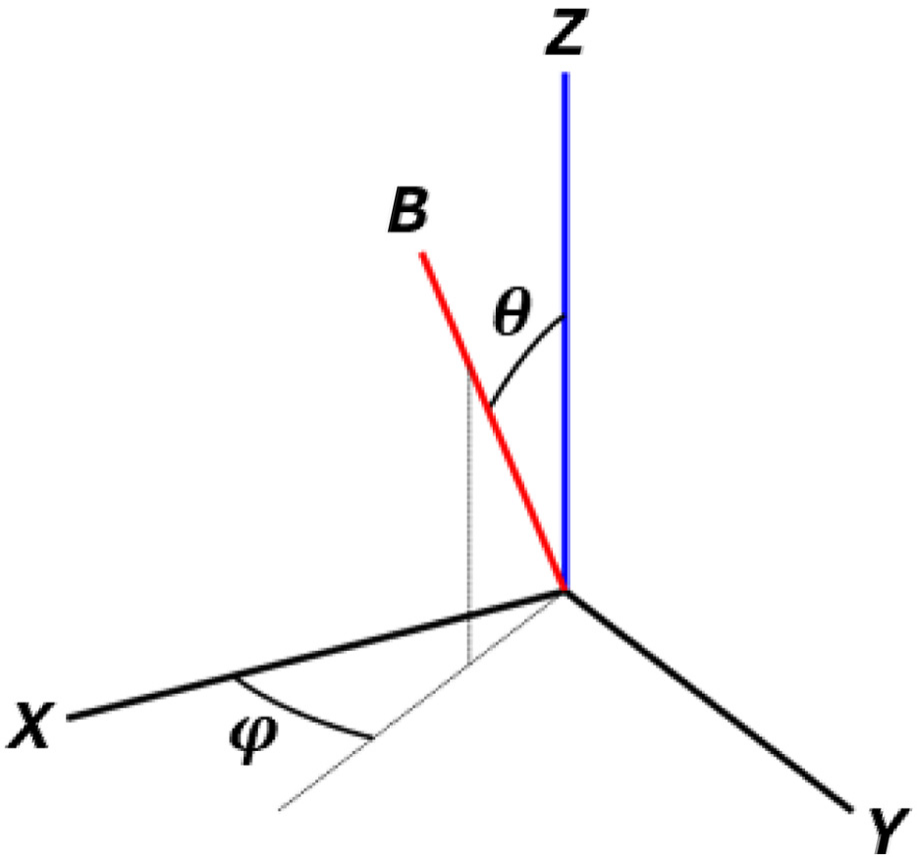
Illustration of DM B (red) in relation to the OCS of reference Z (blue).

A total of nine DMs A, B, C, D, E, F, X, Y, and Z were obtained on the phantom object. DM orientations were varied in space to obtain different levels of NAF (Table 1). Δy was fixed at 0.1 mm. For every DM, two serial measurements were performed with a 6 dB increase in the vibration amplitude between measurements. The shaker, driven at 1 kHz with input voltages 2 and 4 Vpp, resulted in vibration amplitudes of 0.49 and 0.95  mm/s, respectively (Fig. S1 in the Supplementary Material). A, B, C, D, E, F, and Z were used for 13 orthogonal decompositions with Z—optical axis along the vertical—as the reference DM (Fig. 5). An orthogonal decomposition is referred to by three capital letters that correspond to its included DMs, e.g., DM Z, A, and B combine to orthogonal decomposition ZAB. Only orthogonal decompositions with a Δφ≫0  deg were performed, so ZCD and ZEF were excluded.

**Table 1:** Orientation of Phantom direction measurements.

	θ (deg)	φ (deg)
A	40	0
B	30	30
C	20	60
D	40	60
E	10	90
F	40	90
X	90	0
Y	90	90
Z	0	0

All orthogonal decompositions result in a $v_{x_{\varepsilon }}$ and $v_{y_{\varepsilon }}$ (mean of the decomposed components), expressed in the OCS of Z. The direction of the optical axes was varied to illustrate different levels of noise amplification. DM X and Y are DMs with the robot-arm positioned along the Cartesian axes of Z to define velocity components $v_{x}$ and $v_{y}$ (Fig. 5). Considering the robot’s inaccuracy, a more precise estimation of $v_{x}$ and $v_{y}$ was obtained by performing orthogonal decomposition XYZ. The registration error $\varepsilon_{r}$ and noise amplification also apply here, but only very poorly given the near-perfectly conditioned direction matrix. The mean of the decomposed components from orthogonal decomposition XYZ is defined as the ground truths $v_{x}$ and $v_{y}$. The decomposition accuracy was evaluated by calculating $\varepsilon_{d_{x}}=v_{x}-v_{x_{\varepsilon }}$ and $\varepsilon_{d_{y}}=v_{y}-v_{y_{\varepsilon }}$ for all orthogonal decompositions.

### 2.10 Experimental Measurements on the Malleus–Incus Complex

#### 2.10.1 Set-up

The same data-collection protocol as for the phantom experiment was used, except for the stimulus channel. Air conduction stimulation to the ear canal of the specimen was done via an insert phone (ER3•C, Etymotic Research, Illinois, United States), alongside a probe tube microphone (ER7, Etymotic Research, Illinois, United States) inserted through the insert phone plug to measure ear canal sound pressure (ECP) at an estimated 1 to 2 mm from the tympanic membrane. The insert phones were calibrated using a 2-cc coupler (Brüel & Kjær 4152, Denmark), and stimuli were presented at 115 and 113 dB SPL at 1000 and 3000 Hz, respectively. These frequencies were specifically chosen to probe a key property of middle ear mechanics with the setup. It is known that the MIC displays a hinged rotational motion at frequencies below ∼1.5  kHz, with little relative motion between the incus and the malleus. At higher frequencies, other motion components contribute to a more complex vibrational pattern with increased relative ossicular motion.^24^ The stimulus intensities were low enough to prevent any nonlinear distortions occurring in the ossicular chain.^25^

#### 2.10.2 Temporal bone dissection

Measurements were performed on one human fresh frozen temporal bone that was prepared surgically for maximal exposure of the MIC. The temporal bone was provided by the Vesalius Institute (Anatomy and Pathology, University of Leuven—KU Leuven, Belgium) following the ethical approval by the same institute (S69193). Harvesting and use of the temporal bones were conducted following the Helsinki Declaration.

Before the experiment began, the external ear canal and tympanic membrane were inspected for irregularities. Surgical preparation primarily consisted of a standard canal wall-up mastoidectomy complemented with a wide epitympanotomy. To obtain a wide optical access to the MIC, large parts of the surrounding bone and roof, such as the squamous part of the temporal bone, were removed with a bone cutter. In addition, the mastoidectomy was enlarged in the posterior direction with a large drill. After final surgical preparation and before the start of the experiment, the quality of the specimen was verified according to the ASTM F2504-05 guidelines as described by Fierens et al.^26^^,^^27^ (Fig. S3 in the Supplementary Material).

#### 2.10.3 Direction measurements on the MIC

Three DMs were obtained with the robot arm in different positions. The measurement directions were defined by maximizing the angle between the DMs within the optically accessible region. For every DM, an $M_{C}$-scan was constructed according to the method described above. Δy was 0.2 mm. The threshold for background subtraction was fixed at 50 dB. One of three DMs was defined as the reference DM. The custom surface point-cloud volume registration algorithm was used to register the non-reference C-scans to the reference C-scan to estimate the optical axes and define spatially corresponding voxels. After resampling the non-reference $M_{C}$-scans to the reference coordinate grid, the orthogonal decomposition was performed for mutual voxels, expressing the MIC vibrations in the WCS.

To determine the $\sigma_{n}$ of bone tissue, uniform ossicular oscillations were obtained by fixing an isolated ossicle on the shaker (Fig. S4 in the Supplementary Material).

#### 2.10.4 Microcomputed tomography (microCT)

The temporal bone specimen was imaged using a Phoenix Nanotom M MicroCT device (GE Measurement and Control Solutions, Germany), equipped with a tungsten target. The device operated at a voltage of 180 kV and a current of 140  μA. The specimen rotated 360 deg, whereas a total of 1000 images were taken with an exposure time of 500 ms. The microCT-scan had a voxel size of 14.9  μm. The data were processed in Phoenix Datos|x (v2.7.1, Waygate Technologies, Huerth, Germany) using scan optimization and exported as 16-bit.tiff slices. For segmentation, the.tiff slices were loaded into 3D Slicer (v5.6.2) using the ImageStacks extension, taking into account the voxel size of the dataset. The malleus, the incus, the stapes, and the MIC were segmented using 3D Slicer’s grow from seeds functionality.

#### 2.10.5 Expressing the vibrations in the anatomical frame

**Fig. 6:**
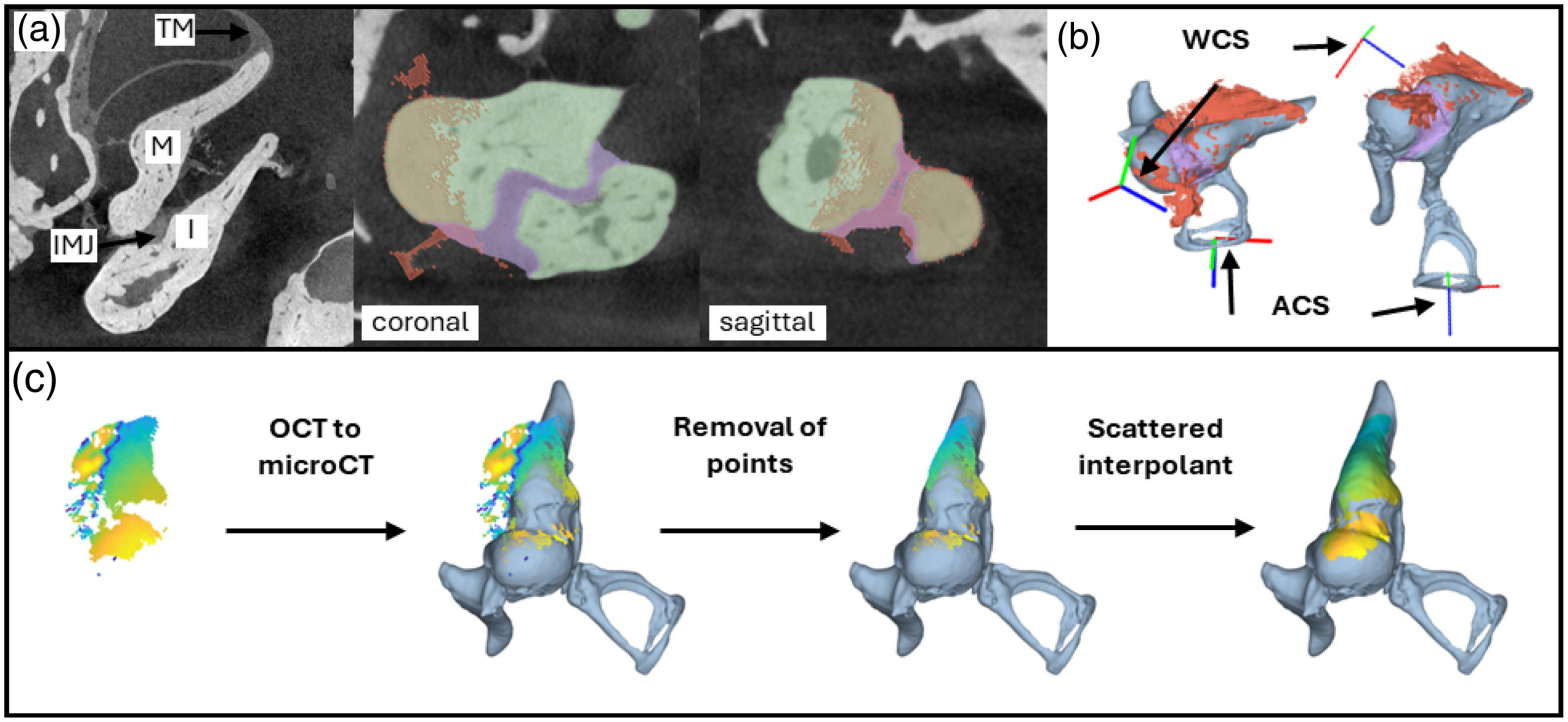
(a) Visualization of a microCT-scan of the middle ear and the OCT to microCT volume registration at different planes through the IMJ (coronal and sagittal). The reference C-scan (red) follows the contours of both the incus and malleus (green) and the IMJ (purple). (b) The anatomically oriented WCS in relation to the ACS in two orientations. In red is the reference C-scan, in grey the malleus, incus and stapes, and in purple, the IMJ. $x^{ACS}$ (red), $y^{ACS}$ (green), and $z^{ACS}$ (blue) define the ACS. (c) Step-wise mapping of the vibratory data in the ACS to the microCT segmentation. Data of $A_{x}^{ACS}$ are shown. TM, tympanic membrane; M, malleus; I, incus; IMJ, incudo-malleolar joint; WCS, world coordinate system; ACS, anatomical coordinate system; $A_{x}^{ACS}$, amplitude component of $v_{x}^{ACS}$; WCS, world coordinate system; ACS, anatomical coordinate system; $A_{x}^{ACS}$, amplitude component of $v_{x}^{ACS}$.

The anatomical coordinate system (ACS) was centered on the stapes footplate and axes were defined as follows: the Z-axis is perpendicular to the stapes footplate, with the positive direction pointing medially, the Y-axis corresponds to the perpendicular projection of the line between the posterior process of the malleus and the umbo onto the Z-axis, with the positive direction of the Y-axis pointing superiorly and the X-axis is perpendicular on the ZY plane with the positive direction posteriorly [Fig. 6(b)]. This ACS was constructed with manually defined and projected fiducials in 3D Slicer. Expression of the vibrations in this ACS required two steps. First, the orientation of the WCS relative to the ossicular chain morphology was assessed by volume registration of the OCT C-scan of the reference DM to the segmented microCT-scan with the custom surface point-cloud volume registration algorithm [Fig. 6(a)]. Second, the MIC vibrations were expressed in the ACS by performing a change of basis from the WCS to the ACS:

$$[\begin{array}{c} v_{x}^{ACS}\\ v_{y}^{ACS}\\ v_{z}^{ACS} \end{array}]=R\times [\begin{array}{c} v_{x}^{WCS}\\ v_{y}^{WCS}\\ v_{z}^{WCS} \end{array}],$$ (17)

where R is the transformation matrix that transforms the anatomically oriented WCS to coincide with the ACS.

#### 2.10.6 Mapping vibrometric data to the microCT segmentation

First, the residual vibratory data were mapped to the surface mesh of the microCT segmentation using the transformation matrix obtained from the OCT to microCT volume registration [Fig. 6(a)]. Next, vibratory data that did not represent the MIC and therefore were located externally from the microCT segmentation were not used for further processing. Only voxels with an amplitude higher than $A_{\min}^{\left(d\right)}$ were retained. Finally, any point of the surface mesh at a distance of <0.1  mm to the closest datapoint was attributed a value. This value was defined with a scattered interpolation method with nearest neighbor interpolation and a boundary extrapolation method [Fig. 6(c)].

## 3 Results

### 3.1 Phantom Experiment

**Fig. 7:**
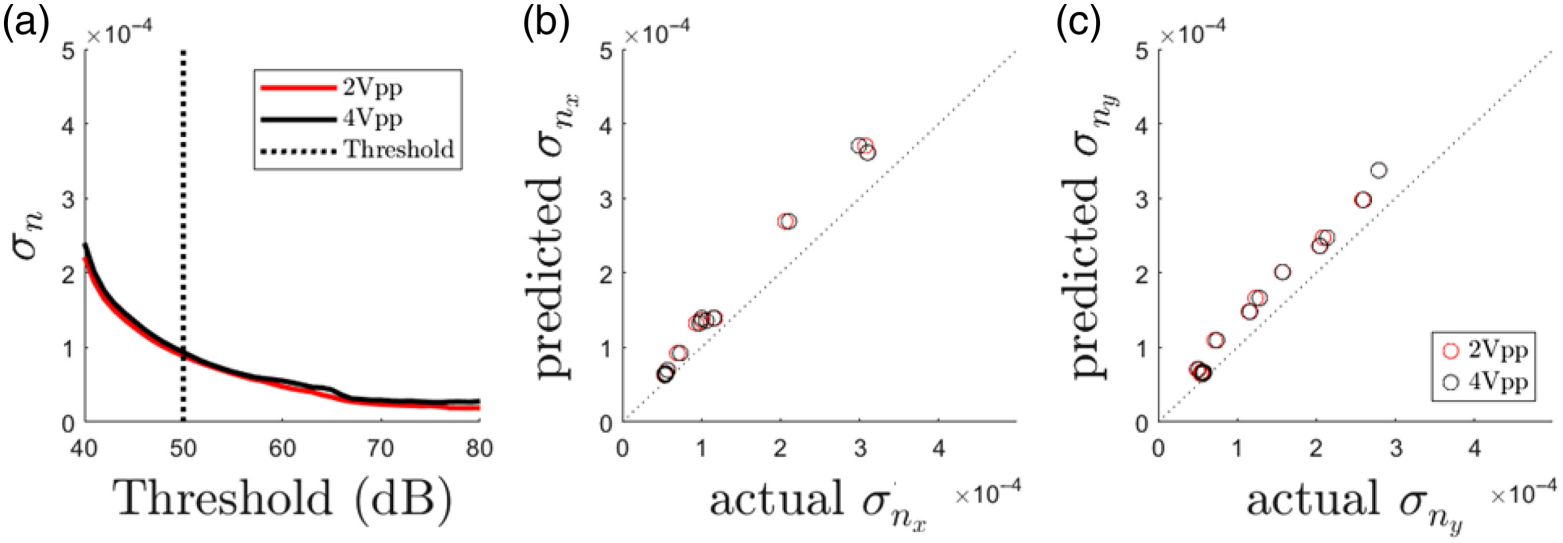
(a) Threshold-$\sigma_{n}$ relation before smoothing due to interpolation. The dotted vertical line is the threshold used for background subtraction. (b), (c) Visualization of the noise distribution in $v_{x}$ and $v_{y}$ after orthogonal decomposition for all decompositions. The prediction is calculated from Eq. (10). The dotted line corresponds to a perfect prediction.

Figure 7 shows the experimental validation of the theoretical framework. The threshold-$\sigma_{n}$ relation that is valid for low SNR measurements at 1 kHz on the phantom object (PMMA) is shown in Fig. 7(a). The two curves that overlap despite a factor 2 increase in vibrational amplitude between measurements are consistent with the amplitude independence of $\sigma_{n}$ at low SNR signals. The dotted vertical line is the threshold of 50 dB used for background subtraction that delivers a good trade-off between sensitivity and data availability. Smoothing due to interpolation yielded a new dataset with a reduced $\sigma_{n}$, from $∼1.0\;\times 10^{-4}$ to $∼0.33\times 10^{-4}$. This corresponds to an $A_{\min}$ of ∼0.1  mm/s [Eq. (13)].

**Fig. 8:**
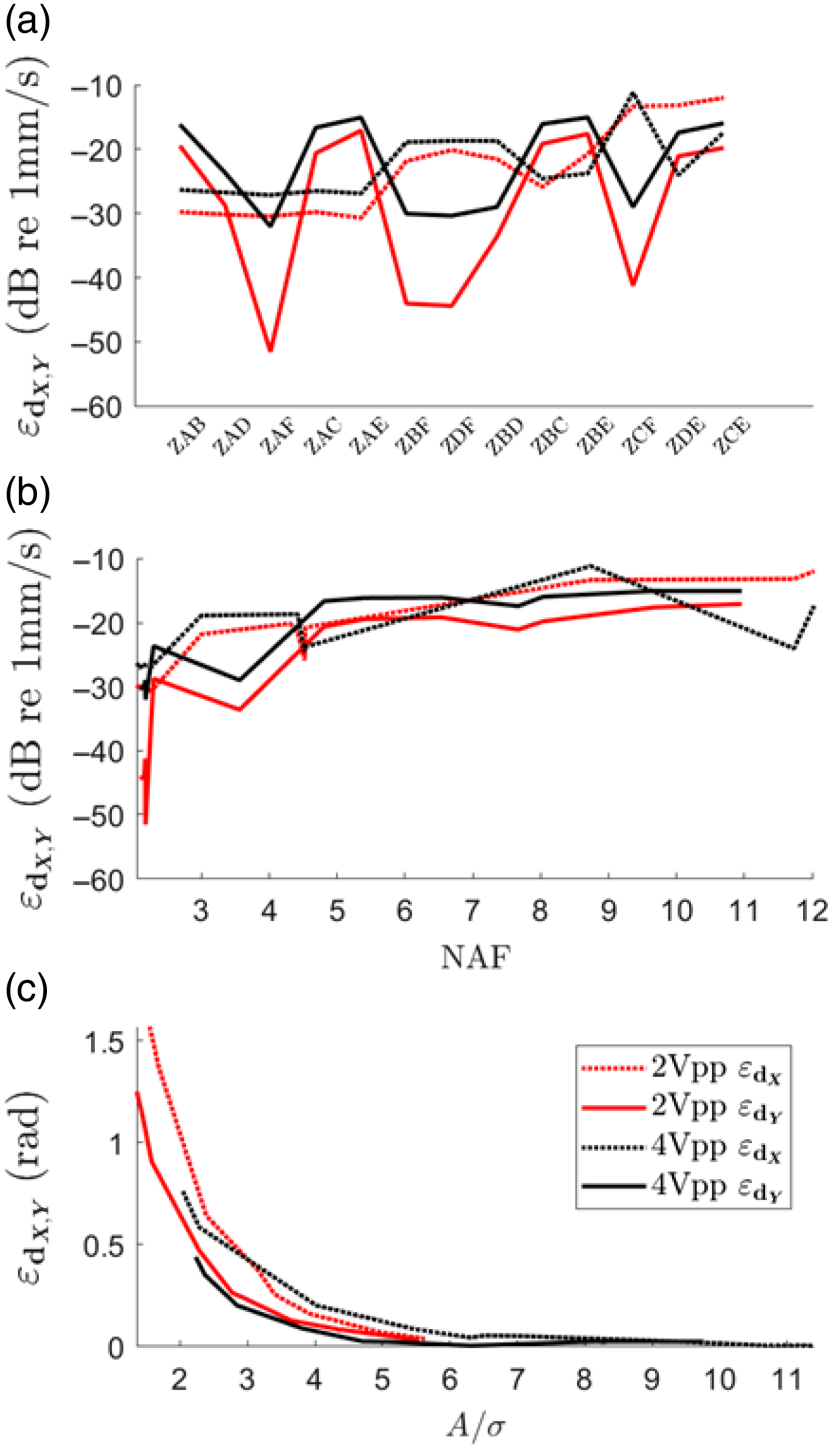
(a) Amplitude components of $\varepsilon_{d_{x}}$ and $\varepsilon_{d_{y}}$ per orthogonal decomposition sorted for increasing $NAF_{x}$. (b) Amplitude components of $\varepsilon_{d_{x}}$ and $\varepsilon_{d_{y}}$ in function of NAF. (c) Phase component of $\varepsilon_{d_{x}}$ and $\varepsilon_{d_{y}}$ in function of $A_{t}^{\left(d\right)}/\sigma_{n}^{\left(d\right)}$.

The predicted $\sigma_{n_{x}}$ and $\sigma_{n_{y}}$ calculated from Eq. (10) scale well with the actual $\sigma_{n_{x}}$ and $\sigma_{n_{y}}$ for all orthogonal decompositions. The slight overestimation of the prediction will result in a slightly higher than actual noise-floor estimation, according to Eq. (15) [Figs. 7(b) and 7(c)]. Figure 8(a) shows the amplitude component of the decomposition errors $\varepsilon_{d_{x}}$ and $\varepsilon_{d_{y}}$ per orthogonal decomposition, sorted for increasing $NAF_{x}$. $\varepsilon_{d_{x}}$ and $\varepsilon_{d_{y}}$ can be very different within the same orthogonal decomposition, e.g., ZDF results in a low $\varepsilon_{d_{y}}$ but a high $\varepsilon_{d_{x}}$. Interestingly, according to Eq. (6), the amplitude component of the decomposition error increases with the vibration amplitude. In Fig. 8(b), the same amplitude components of $\varepsilon_{d_{x}}$ and $\varepsilon_{d_{y}}$ are plotted as a function of NAF. As described above, the amplitude component of $\varepsilon_{d_{x}}$ and $\varepsilon_{d_{y}}$ increases with NAF, but in an unpredictable manner due to the impact of the orientation of the erroneous optical axis on the amplitude decomposition error.

More importantly, at a high NAF level, the noise contained in the decomposed components is amplified to the extent that $A_{t}^{\left(d\right)}/\sigma_{n}^{\left(d\right)}<3$, i.e., a Rician bias is introduced. At this stage, not only does the increased sensitivity to misalignments contribute to the amplitude component of $\varepsilon_{d_{x}}$ and $\varepsilon_{d_{y}}$, but also the rectification of the low SNR signal. This explains why at a NAF greater than approximately five, the amplitude component of $\varepsilon_{d_{x}}$ of the 2Vpp measurement approaches and surpasses that of the 4Vpp measurement.

On the other hand, the phase component of $\varepsilon_{d_{x}}$ and $\varepsilon_{d_{y}}$ is more stable [Fig. 8(a)]. According to Eq. (16), an increase of $A_{t}^{\left(d\right)}/\sigma_{n}^{\left(d\right)}$ corresponds to a decrease in $\sigma_{\varphi }^{\left(d\right)}$ and therefore a more accurate estimation of the true decomposed phase $\varphi_{t}^{\left(d\right)}$. At the noise floor $A_{t}^{\left(d\right)}/\sigma_{n}^{\left(d\right)}=3$, a significant phase error of 0.25 rad is present. However, at higher SNR signals with $A_{t}^{\left(d\right)}/\sigma_{n}^{\left(d\right)}>5$, the residual phase error becomes negligible.

### 3.2 MIC Measurements

For a threshold of 50 dB, $\sigma_{n}$ was $0.95\times 10^{-4}$, which is comparable to the phantom object’s $\sigma_{n}$. Interpolation gives a similar smoothing of the dataset so that also for the *ex vivo* experiments $A_{\min}$ is approximately equal to 0.1  mm/s. The polar and azimuthal angles of the two non-reference DMs used for decomposition were 20 deg and −143  deg for $d^{2}$ and 45 deg and −177  deg for $d^{3}$. Consequently, $NAF_{x}$ and $NAF_{y}$ were 3.87 and 2.03, respectively. This results in an estimated amplified noise floor $A_{\min}^{\left(d\right)}$ of 0.39  mm/s for measurements at both 1 and 3 kHz.

**Fig. 9:**
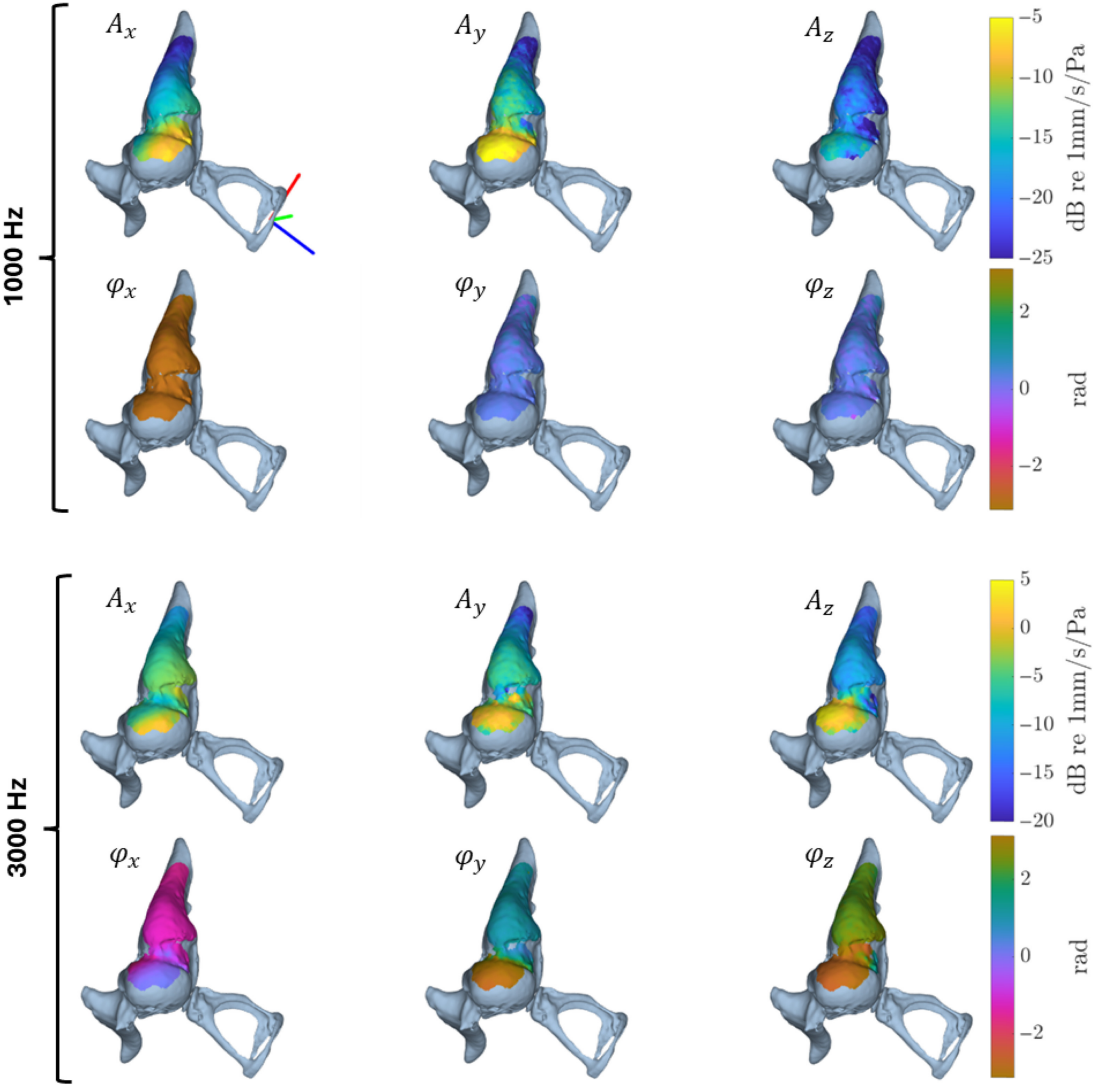
Vibrometric data mapped onto the microCT-segmentation. The upper panel corresponds to the 1000 Hz stimulation, the lower panel to the 3000 Hz stimulation. For both, the first row corresponds to the vibration amplitude and the second row to the vibration phase. The ACS is added to the first ossicular chain visualization ($x^{ACS}$ in red, $y^{ACS}$ in  green, and $z^{ACS}$ in blue). Note the different color scale for the vibration amplitudes.

Figure 9 shows the amplitudes normalized for the ear canal pressure and phases of the MIC expressed in the ACS mapped onto the microCT segmentation for an acoustic stimulus of 1000 and 3000 Hz at 115 and 113 dB SPL, respectively. At 1000 Hz, a hinge-like motion occurs, with higher velocities at the malleus head. The phase maps show an MIC that is entirely in phase. The dominant motion is directed along the Y-axis. At 3000 Hz, the MIC shows overall increased velocities. In addition, a more complex vibrational pattern arises, with a twist component that complements the hinge-like motion. This is shown by the phase shift that gradually emerges in the incudo-malleolar joint as visualized in the bottom row of Fig. 9. Finally, the dominant motion shifts toward the Z-axis.

## 4 Discussion

Investigating the 3D nanometer vibrations of the human ossicular chain demands a versatile and integral detection method, mainly due to the limited optical access that can be surgically achieved and the relatively large structures that must be imaged. Here, a method is shown with a commercially available, robot-mounted OCT scanning system capable of serial 3D vibrometric measurements. To our knowledge, this is the first single-beam OCTv setup incorporating a rotational component that integrates 3D structural imaging with 3D motion analysis.

Previous studies on 3D motion of the middle ear used LDV to measure vibrations at multiple discrete points along the surface of the ossicular chain. Although rigid-body models can be applied to derive 3D middle ear motion components, they inherently ignore shear deformation.^24^^,^^28^

The current method with background subtraction overcomes this limitation. The optically accessible region of the MIC is almost continuously mapped, independent of local tissue properties. To integrally model the vibrational patterns of the ossicular chain, the *ex vivo* measurements presented here can be expanded with additional 3D OCTv measurements. These can be combined, even across different acquisition sessions, and “patched” onto the structure (Fig. 9). Optically inaccessible regions can be attributed a value through extrapolation or by fitting a finite-element model with mechanical parameters. This method eliminates the need to support on the rigid-body assumption, which can be either validated or challenged by future research.

Another key advantage of the method is that the morphology captured in C-scans enables estimation of the optical axes of different DMs without the need for external calibration. On a phantom object, the estimated registration error was just 0.4 deg, which is comparable to or better than other multidimensional vibrometry approaches.^10^^,^^15^^,^^16^ Although the estimated performance of the volume registration algorithm depends on the morphological features contained in the phantom object, the registration accuracy on ossicular geometries can be expected to be similar and certainly suited for mechanical modeling of the middle ear.

With the presented theoretical framework, we have illustrated the complex interplay between the sensitivity of the orthogonal decomposition to optical axis misalignment and to noise amplification, which possibly leads to amplitude overestimation due to Rician bias. The NAF was identified as a key factor to manage both factors and maintain decomposition accuracy. First, no Rician bias is introduced to the decomposed amplitude components in regions exceeding the amplified noise floor $A_{\min}^{\left(d\right)}$. Second, the misalignment sensitivity does not result in an exponential amplitude decomposition error increase up to a NAF of 12—higher levels were not analyzed as we do not expect such experimental circumstances. In addition, the amplitude decomposition error scales with amplitude: a 6 dB increase in vibration amplitude results approximately in a 6 dB increase in the amplitude decomposition error. This scaling is inherent to Eq. (6) where the systemic error in the direction matrix, introduced by erroneous optical axis estimation, can be magnified by multiplication with the (noisy) measured components. A greater displacement amplitude results in a higher multiplication of the systemic error and consequently a greater decomposition error. This implies that sample vibrations should be kept within reasonable limits. On the other hand, the phase decomposition improves with vibration amplitude and is already reliable at low amplitudes, as shown for $\varphi_{z}$ at 1 kHz where amplitudes are only slightly above the noise floor (Fig. 9).

The results of the *ex vivo* experiment demonstrate that our approach is highly suited for research in middle ear mechanics. The smoothing due to interpolation is undesirable when analyzing vibratory patterns of small structures that extend over only a few pixels but beneficial for research applications focused on relatively large, (semi-)rigid structures. The orientation of the optical axes in the *ex vivo* experiment, and the corresponding $NAF_{\text{max}}$, is representative for 3D OCTv measurements on the MIC. However, some regions along the ossicular chain will only be optically accessible through a smaller window, necessitating smaller degrees of angulation between DMs and thereby increasing $NAF_{\max}$. If, however, the decomposed vibration amplitudes fall below the amplified noise floor $A_{\min}^{\left(d\right)}$, one can opt to increase the reflectivity threshold for background subtraction, thereby decreasing $A_{\min}^{\left(d\right)}$ at the cost of sub-surface information.

Although the vibratory responses at the tissue surface are shown, sub-surface motion analysis requires further refractive correction to accurately define the spatial correspondence of sub-surface voxels. In addition, because thresholding results in sparse subsurface data, advanced signal processing techniques should be applied beyond the voxel-to-voxel orthogonal decompositions proposed here. Future work should investigate surface and subsurface 3D shear components in the ossicles, ossicular joints, and suspensory ligaments. These insights will be crucial to fully understand the mechanisms of acoustic energy transmission through the ossicular chain, which can contribute to the optimization of middle ear implants, such as actuators, microphones, or prostheses.

## 5 Conclusion

A robot-mounted 3-D OCTv set-up is presented that introduces a rotational element directly into the scanning system, offering a new approach to multidimensional OCTv. A volume registration method was developed that enables the estimation of the optical axis with an accuracy of 0.4 deg. In addition, we proposed a theoretical framework to estimate both the amplified noise floor following orthogonal decomposition and the accuracy of the decomposed components, depending on the relative orientation of the optical axes. Future research should aim to integrally map the ossicular chain vibrations and focus on the sub-surface vibratory responses to critically assess the rigid-body assumption.

## Supplementary Material

10.1117/1.JBO.31.12.123303.s01

## Data Availability

The data that support the findings of this article are not publicly available due to privacy concerns. They can be requested from the author at nicolas.verhaert@kuleuven.be.
