# Supplementary material for: 3D-motion mapping of the malleus–incus complex using a robot-mounted optical coherence tomography vibrometry system
Source: J Biomed Opt. 2026 Feb 20;31(12):123303. doi: 10.1117/1.JBO.31.12.123303 (PMC12935278; doi:10.1117/1.JBO.31.12.123303)
Supplement: Supplementary file 1 [file JBO_031_123303_SD001.pdf]

Supplemental: 3D-Motion Mapping of the Malleus-Incus Complex using a Robot-Mounted Optical Coherence Tomography Vibrometry System

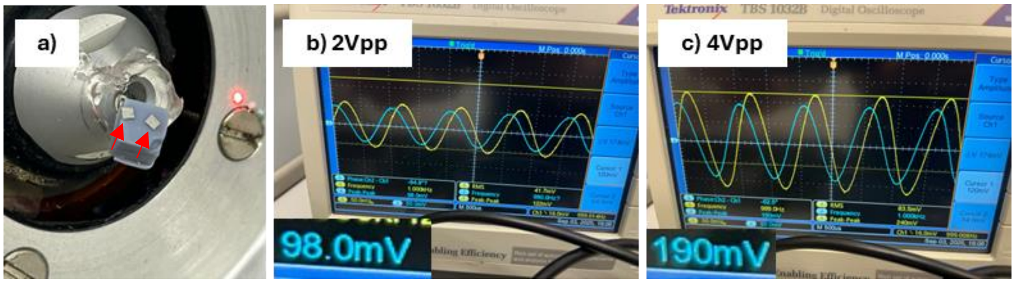

Fig. S2 LDV validation of true vibration amplitude A_t

(a) A reflective tape was applied at two positions on the upper horizontal surface of the 3D printed object (red arrows). (b-c) Two measurements were performed with the LDV's optical axis along the vertical for each vibration intensity (shaker driven at 2Vpp and 4Vpp). The sensitivity of the shaker was 10mm/s/V. Velocity and phase were equal at the two measurement points, confirming the uniform oscillation of the object.

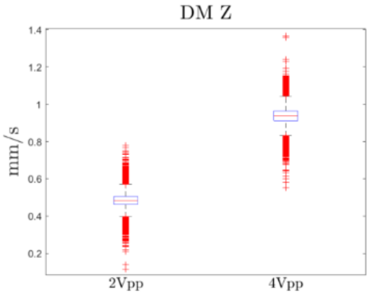

At 2Vpp and 4Vpp , the LDV-measured velocity corresponds to $10mm/s/V * 0.098/2V = 0.49mm/s$ and $10mm/s/V * 0.190/2V = 0.95mm/s$, respectively. This corresponds to the amplitude measured via the OCT measurements along the vertical (DM Z, boxplots) that had a mean amplitude of 0.484mm/s and 0.933 mm/s, respectively. The deviation between the LDV and OCTv results can be attributed to a slight misalignment of the LDV optical axis and OCTv optical axis, as we cannot calibrate both axes to be exactly equal.

Fig S2 Volume registration accuracy

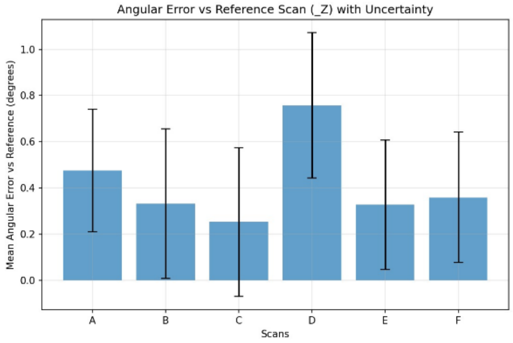

Fig. S2 shows the averaged results of the plane-based registration algorithm, for each non-reference volume registered to the reference volume Z with 30 slightly varied point cloud resampling parameters (voxel size was varied between 29 and 31 microns). The results show a mean registration error ε_r of 0.42° with a standard deviation of 0.17°. Error bars indicate the angular uncertainty of the fitted planes, which is contributed by the noise in the C-scans. The uncertainty is estimated to be on average 0.30°, by resampling the plane points and comparing the variations of plane angles.

Fig. S3 Middle ear transfer function

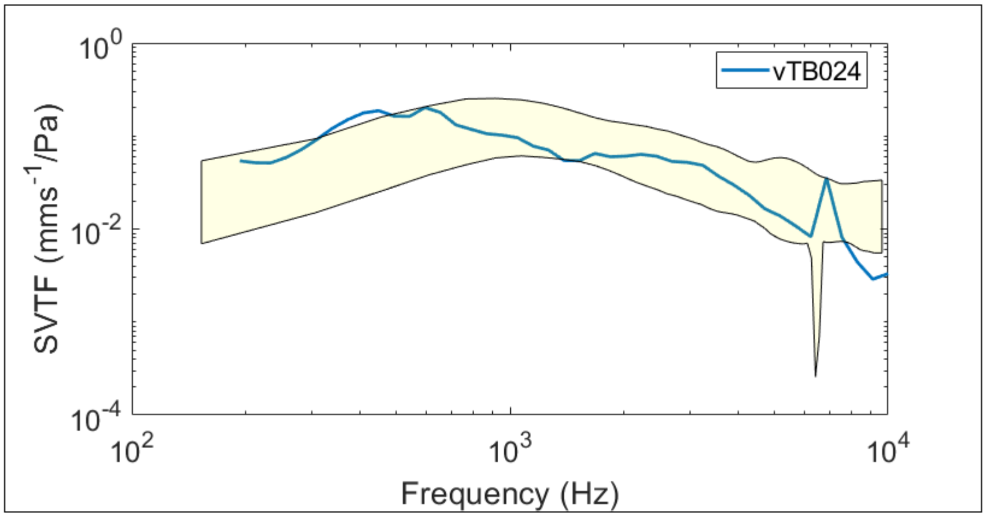

The middle ear transfer function was evaluated after final surgical preparation according to the method described in Fierens et al. In yellow the Rosowski-criteria and in blue the middle ear transfer function of the temporal bone specimen.

Fig. S4 validation of uniformity of ossicular oscillations

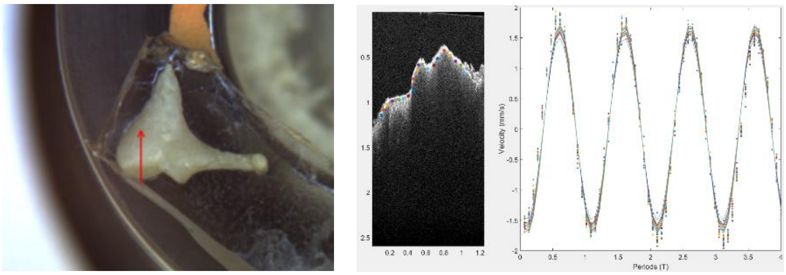

Validation of the uniformity of ossicular oscillations for the estimation of σ_n on bone tissue. An isolated incus is fixed on the shaker with double-side tape. Velocity points along the cross-section show similar oscillations. The slight variation can be attributed to the measurement noise in this unfiltered data.
